# Population‐level variation in parasite resistance due to differences in immune initiation and rate of response

**DOI:** 10.1002/evl3.274

**Published:** 2022-02-24

**Authors:** Amanda K. Hund, Lauren E. Fuess, Mariah L. Kenney, Meghan F. Maciejewski, Joseph M. Marini, Kum Chuan Shim, Daniel I. Bolnick

**Affiliations:** ^1^ Department of Ecology, Evolution, and Behavior University of Minnesota St. Paul Minnesota 55123; ^2^ Department of Ecology and Evolutionary Biology University of Connecticut Storrs Connecticut 06269; ^3^ Current Address: Department of Biology Texas State University San Marcos Texas 78666; ^4^ Department of Ecology, Evolution, and Behavior University of Texas at Austin Austin Texas 78712

**Keywords:** Fibrosis, immune evolution, immune response, parasite resistance, *Schistocephalus solidus*, threespine stickleback

## Abstract

Closely related populations often differ in resistance to a given parasite, as measured by infection success or failure. Yet, the immunological mechanisms of these evolved differences are rarely specified. Does resistance evolve via changes to the host's ability to recognize that an infection exists, actuate an effective immune response, or attenuate that response? We tested whether each of these phases of the host response contributed to threespine sticklebacks’ recently evolved resistance to their tapeworm *Schistocephalus solidus*. Although marine stickleback and some susceptible lake fish permit fast‐growing tapeworms, other lake populations are resistant and suppress tapeworm growth via a fibrosis response. We subjected lab‐raised fish from three populations (susceptible marine “ancestors,” a susceptible lake population, and a resistant lake population) to a novel immune challenge using an injection of (1) a saline control, (2) alum, a generalized pro‐inflammatory adjuvant that causes fibrosis, (3) a tapeworm protein extract, or (4) a combination of alum and tapeworm protein. With enough time, all three populations generated a robust fibrosis response to the alum treatments. Yet, only the resistant population exhibited a fibrosis response to the tapeworm protein alone. Thus, these populations differed in their ability to respond to the tapeworm protein but shared an intact fibrosis pathway. The resistant population also initiated fibrosis faster in response to alum, and was able to attenuate fibrosis, unlike the susceptible populations’ slow but longer lasting response to alum. As fibrosis has pathological side effects that reduce fecundity, the faster recovery by the resistant population may reflect an adaptation to mitigate the costs of immunity. Broadly, our results confirm that parasite detection and immune initiation, activation speed, and immune attenuation simultaneously contribute to the evolution of parasite resistance and adaptations to infection in natural populations.

**Impact Summary**

Dramatic variation in resistance to parasites is common within and among populations of hosts. Yet the mechanisms underlying these evolved differences remain unclear. Many evolution studies focus on the broad outcomes of infection (infected or not) when studying resistance, without specifying what part of the immune response has evolved. Here, we experimentally partition different sequential stages in the host immune response (initiation, actuation, attenuation) to evaluate which stage(s) underly the evolution of host resistance to infection. This study compares three populations of threespine stickleback that naturally differ in their ability to resist infections and suppress the growth of a freshwater tapeworm. These include a “resistant” lake population, a “susceptible” lake population, and an “ancestral” marine population that is rarely exposed to the tapeworm in nature but is susceptible when exposed in the lab. The resistant population exhibits a fibrosis immune response to infection, which has been linked to suppressed tapeworm growth and viability. We injected different immune challenges directly into the site of infection (peritoneal cavity) and measured the subsequent fibrosis response through time. We found that all populations were capable of producing fibrosis in response to a general immune stimulant (alum). But only the resistant population was able to recognize and respond to tapeworm protein alone. This population also responded faster than the others, within 24 hours, and attenuated its fibrosis by 90 days post injections, whereas the other populations exhibited a slower response that did not attenuate in the study timeframe. We concluded that, in this system, rapid evolution of parasite resistance entails changes in initiation of an immune response, rather than gain or loss of the ability to perform that response.

The co‐evolutionary arms race between hosts and parasites can shift rapidly across space and time (Karvonen and Seehausen 2012; Fernandes et al. 2019), generating stochastic or deterministic variation between isolated populations (Papkou et al. 2016). Indeed, many closely related populations vary in their ability to resist infection, even to the same parasite (Roy and Kirchner 2000; Boots et al. 2009; Vale et al. 2011). Although this is a common pattern, our understanding of the underlying mechanisms that drive such rapid evolution of parasite resistance is still limited.

Most evolutionary studies in wild populations focus on the broad outcomes of infection, such as infection rates, resistant/susceptible phenotypes, or parasite load. However, infection outcomes depend on a series of sequential and stepwise interactions between hosts and parasites (Hall et al. 2017). Understanding where in this chain of events variation occurs is essential to understanding what selective pressures are shaping host‐parasite evolution. For example, ecological or behavioral factors can influence the degree to which hosts are exposed to parasites (Stutz et al. 2014; Barron et al. 2015). Once infected, resistance depends on the host's ability to (1) detect the presence of a parasite, (2) initiate a suitable immune response, (3) actuate that response at a level that will eliminate or control the parasite, then (4) attenuate that response (Hall et al. 2017). Each of these steps can be costly, such as the energetic cost of initiating and maintaining an activated state (Ganeshan and Chawla 2014), the risk of auto‐immune responses (recognition), oxidative stress from the effector response (Viney et al. 2005), or the risk of continued infection. Therefore, hosts should avoid initiating immune responses unnecessarily, and when activated, must be able to modulate this response at the appropriate time, not prematurely, but also not too late as to impose excess cost once the danger has passed (Khan et al. 2017; Armour et al. 2020). Variation at any of these steps can lead to differences in infection outcomes and immunopathology side effects. Indeed, each step influences selection on subsequent steps and likely entails different genes, selective pressures, costs, and opportunities for parasite counteradaptation. Thus, breaking down and isolating different steps of the infection cycle is crucial for understanding the evolution of parasite resistance (Hall et al. 2017, 2019). By experimentally partitioning different components of the host response to infection through time, we provide a detailed look at the evolution of resistance in populations of threespine stickleback that vary in their response to the tapeworm, *Schistocephalus solidus*.

The threespine stickleback has become a model for studying local adaptation because of their repeated colonization of freshwater lakes from the ancestral marine population (∼11,000 years ago in British Columbia) (Bell and Foster 1994). Marine populations persist largely unchanged (Bell and Foster 1994; Roberts Kingman et al. 2021; Kirch et al. 2021), allowing us to infer the ancestral character state, and genotypes, of the initial freshwater colonists. Because *S. solidus* eggs do not hatch in brackish water (Simmonds and Barber 2016), marine fish are rarely exposed, and *S. solidus* represented a new challenge for fish invading freshwater lakes. Infections of *S. solidus* can be quite costly for fish: decreasing antipredator responses (Giles 1983; Milinski 1985), swimming ability (Blake et al. 2006), body condition and energy reserves (Tierney et al. 1996; Barber and Svensson 2003), and investment in reproduction (Schultz et al. 2006), although these effects can vary by population (MacNab et al. 2009). These costs favored the parallel evolution of partial resistance to *S. solidus* in numerous freshwater populations (Weber et al. 2017b), despite some immune suppression by the tapeworm (Scharsack et al. 2004).

Some lake populations exhibit a particularly effective form of resistance, involving tapeworm‐induced formation of extensive fibrotic tissue throughout the peritoneal cavity where infections occur (Lohman et al. 2017; Weber et al. 2017b, 2021; De Lisle and Bolnick 2021). Other lake populations do not exhibit fibrosis when infected and are both more readily infected and permit faster tapeworm growth. Quantitative Trait Locus (QTL) mapping of tapeworm‐exposed lab‐raised F2 hybrids between high‐ and low‐infection lakes confirms that fibrosis contributes to tapeworm growth suppression, and the formation of granulomas around small tapeworms that frequently kill the parasite (Weber et al. 2017b, 2021). Field surveys on Vancouver Island confirm these lab results: both within and among lakes, fibrosis is associated with reduced tapeworm infections, smaller tapeworms, and more encysted dead tapeworms (Weber et al. 2021). Fibrosis rarely occurs spontaneously in naïve laboratory stickleback without an immune challenge (pers. obs.). Fibrosis is also not observed in response to other peritoneal tissue‐encysting parasites such as the nematode *Eustrongylides* sp. (Weber et al. 2021). Thus, multiple lines of evidence point to fibrosis as an adaptation, in some lakes, to suppress tapeworm survival or growth. However, fibrosis is also a pathology: both females and males are less likely to reproduce when they have fibrosis (controlling for infection status) and fibrotic females produce fewer eggs (De Lisle and Bolnick 2021; Weber et al. 2021). Given this mix of costs and benefits, evolution should act to minimize unnecessary initiation of fibrosis, while also maximizing the rapid onset of fibrosis against infection, and the rate of clearance after the infection has passed.

To understand how selection on and variation in different aspects of the fibrosis response may be generating among‐population differences in parasite resistance, we need to tease apart where in the infection cycle variation is arising. To do this, we focused on three populations: (1) a “resistant” lake population with common and severe fibrosis and small tapeworms, (2) a “susceptible” lake population with abundant large tapeworms and negligible fibrosis, and (3) an “ancestral” marine population with negligible exposure to the tapeworm in nature, high susceptibility to laboratory infections, and negligible fibrosis (Weber et al. 2017a). We first performed a survey in both lake populations to confirm population‐level differences in the presence of fibrosis. We then performed a laboratory experiment that isolated different stages of the host response using different injected immune challenges. Our experimental design allows us to test several hypotheses for why parasite resistance varied among these populations: (H1) variation in resistance is driven by ecological factors (i.e., lake differences) and all populations will mount similar responses to immune challenges in a common garden laboratory setting, (H2) populations differ in their ability to initiate fibrosis following exposure to tapeworm antigens (either because of differences in detection or early regulatory triggers), and (H3) populations differ in their capacity to actuate a robust peritoneal fibrosis response in general.

If H2 were to be supported (populations differ in whether they initiate a shared fibrosis trait), we were also interested in testing whether there was variation in the timing of initiating or resolving that response. We predicted that the resistant population may have evolved the ability to respond rapidly, to limit the growth of the tapeworm early in infection. The resistant population may also be faster to recover from fibrosis, to mitigate immunopathological costs.

## Methods

### STUDY SYSTEM

*Schistocephalus solidus* has a complex life cycle where it is trophically transmitted from copepods to threespine sticklebacks to birds (Orr et al. 1969; Barber and Scharsack 2010). When a fish consumes an infected copepod, the tapeworm penetrates the intestinal wall and enters the peritoneal cavity. It then grows rapidly and becomes capable of reproducing within its definitive host when it crosses a threshold of ∼50 mg (Tierney and Crompton 1992; Barber et al. 2004). This size threshold corresponds with an increase in the costs associated with tapeworm infections, including behavioral manipulation that likely increases the probability of bird predation (Barber and Scharsack 2010). Growth suppression by the host thus will serve to both prevent parasite reproduction and mitigate infection symptoms, although the infection itself usually persists, presenting a gray area between resistance and tolerance. Phylogeographic studies of *S. solidus* suggest isolation by distance and relatively fine‐scale population structure that is influenced by both lake and year (Stefka et al. 2009; Sprehn et al. 2015).

### FIELD SURVEY AND BREEDING

The following field collections were approved by the Ministry of Forests, Lands, Natural Resource Operations and Rural Development (Collection Permit NA19‐457335). The sites were all within the historical tribal region of the Kwakwaka'wakw First Nation. Methods were approved by the University of Connecticut IACUC (protocol A18‐008).

Sayward Estuary (ancestral population, 50°22ʹ46ʺN, 125°56ʹ43ʺW) is a breeding location for the anadromous marine stickleback (see map, Fig. S1). Sayward fish spend the majority of their life at sea and have very little natural exposure to *S. solidus*. They are highly susceptible in laboratory infection experiments, consistently producing large tapeworms with negligible fibrosis (Weber et al. 2017a).

Fish in Gosling Lake (susceptible population, 50°03ʹ47ʺN, 125°30ʹ07ʺW) are also rarely found to have fibrosis in nature, despite a high prevalence of infection with large tapeworms (50–80% in field surveys from 2004 to present [Weber et al. 2017b, 2021]). In laboratory infections, Gosling fish are less susceptible compared to marine fish, but exhibit no fibrosis and permit rapid tapeworm growth when infected (Weber et al. 2021). Gosling fish may have evolved a tolerance strategy, judging by a genomic evidence of positive selection favoring loss‐of‐function deletions in pro‐fibrosis genes (Weber et al. 2021).

Roselle Lake (resistant population, 50°31ʹ13ʺN, 126°59ʹ12ʺW) has a high incidence of fibrosis in nature with small tapeworms. The presence of granulomas (often containing small dead tapeworms) indicates that these fish can successfully clear some infections and suppress tapeworm growth when they do establish. Roselle has a low to intermediate infection prevalence, with 7–40% of fish infected during field surveys depending on the year (De Lisle and Bolnick 2021; Weber et al. 2021). The fibrosis, small tapeworms, and granulomas that we observe in Roselle are consistent with a “resistance” phenotype seen in other lake populations and experimentally confirmed with laboratory infections (Weber et al. 2021). Roselle and Gosling lakes are in separate watersheds (∼150 km away) and contain isolated stickleback populations with no access to the ocean and limited capacity for gene flow due to inhospitable outlet and inlet streams. Past research on Vancouver Island has indicated clear genomic differentiation between watersheds (Stuart et al. 2017), and even between different lakes within the same watershed (Caldera and Bolnick 2008).

In the spring of 2018, we sampled 31 uninfected and 31 infected fish from Gosling Lake and 30 uninfected and 32 infected fish from Roselle Lake using minnow traps to quantify average tapeworm size and the frequency and severity of fibrosis. Fish were sampled as part of a larger study where we sampled the first 30 uninfected fish and then continued sampling until we had found 30 infected fish for each population. Fish were categorized as uninfected if we did not find a living tapeworm. We scored fibrosis in the peritoneal cavity visually using a dissecting microscope as 0 (no fibrosis), 1 (some fibrosis, organs do not move freely), 2 (fibrosis adhering organs together), 3 (organs adhered together and to the peritoneal wall), and 4 (severe fibrosis, difficult to open peritoneal cavity) (see fibrosis scoring video in the Supporting Information).

We weighed tapeworms on a digital scale; tapeworms weighing less than 0.01 g were recorded as <0.01 g (limit of our field scale) and were entered as 0.009 g for summary statistics. If fish were infected with multiple tapeworms, we weighed all tapeworms together to get average parasite mass. We compared infection intensity between lakes using a general linear model (glm) with a Poisson distribution, and average tapeworm mass using a glm with a gamma distribution and inverse link function. We also compared the number of tapeworms above and below the threshold of our field scale per lake using a chi‐square test. We compared the fibrosis scores of uninfected and infected fish between lakes using Mann‐Whitney *U* tests. To get an additional estimate of infection prevalence for Roselle, we euthanized and preserved 169 randomly selected fish in ethanol, which were later dissected and scored as infected or uninfected (ethanol preservation is not conducive to scoring fibrosis).

In June 2018, we collected fish from our three populations for breeding. Using standard in vitro fertilization methods, we created full‐sibling families from each population and transported fertilized eggs to the lab for rearing (Divino and Schultz 2014). Fish were in two rooms at the animal care facility of the University of Connecticut. Families were often, although not always, split across multiple tanks located in both rooms. All fish were ∼11 months old when they were injected with different immune challenges in May 2019.

### LABORATORY INJECTION EXPERIMENT

We injected four different inoculants directly into the peritoneal cavity. These included (1) 20 μL of 1× phosphate‐buffered saline (PBS, control treatment), (2) 10 μL of homogenized tapeworm protein solution + 10 μL PBS (tapeworm treatment), (3) 10 μL of Alum (2% Alumax Phosphate, OZ Bioscience) + 10 μL PBS (alum treatment), and (4) 10 μL tapeworm protein + 10 μL Alum (tapeworm + alum treatment). Alum is an immune adjuvant that causes the recruitment of leukocytes that initiate an immune response (Kool et al. 2012). Pilot studies demonstrated that alum injections could induce a fibrosis response in the peritoneal cavity of stickleback (Dr. Natalie Steinel, per. comm.). The tapeworm protein solution was used to test if fish could recognize and respond to tapeworm antigens. By using homogenized tapeworms, our experimental design mitigates active interference by the tapeworm, which are well known to secrete a suite of immunomodulatory molecules that suppress and shift host immune responses (Coakley et al. 2016; Maizels et al. 2018; Motran et al. 2018), including in stickleback (Scharsack et al. 2013).

To create this solution, we used tapeworms collected from Farwell Lake (50°11ʹ60ʺN, 125°35ʹ27ʺW) in 2008 that were flash frozen and stored at −80°C. We chose tapeworms from a different lake, watershed, and year to minimize any localized genetic structure of the parasite that may influence population level responses. Tapeworms were dipped in deionized water and placed in chilled 0.9× PBS. Each tapeworm was sonified on ice twice for 1 min (Branson 150 Ultrasonic Cell Disruptor, level 5). Between sonification rounds, samples were chilled on ice (5 min). The homogenized solutions were centrifuged (4°C, 4000 rpm, 20 min) and the supernatant was collected and pooled. We measured the protein concentration using a Red 660 kit (G‐Biosciences), diluted the solution to 1 mg/mL using 0.9× PBS, and stored it at −20°C.

Before injection, fish were lightly anesthetized using a neutral‐buffered MS‐222 (50–75 mg/L). We used ultrafine syringes (BD 31G 8 mm) to inject 20 μL into the lower left side of the peritoneal cavity, slightly above where the end of ventral spine rests. Injections were shallow and at an angle parallel to the body to avoid injuring organs. We watched for visual distention of the peritoneal cavity to ensure solutions were being injected correctly. Solutions were prepared and syringes were loaded in a sterile culture hood. Fish were also given a small colored elastomer mark (Northwest Marine Technologies) corresponding to their treatment group injected subcutaneously just posterior to the neurocranium. Although injecting elastomers can impact immune responses (Henrich et al. 2014), all fish received the same amount of elastomer in the same location across treatment groups. During the injection procedure, fish were placed on a wet sponge and had their gills covered with a wet paper towel. In total, the procedure lasted less than 1 min, and fish were immediately placed in an aerated recovery tank before being returned to their home tank with negligible mortality and no noticeable adverse effects.

We euthanized fish to measure fibrosis post injection at four timepoints: 1, 10, 42, and 90 days. These timepoints were chosen to see (1) if fish could respond within 24 hours to an immune challenge, which is when the tapeworm penetrates the gut (Hammerschmidt and Kurtz 2007) and (2) how their response changed through time as the tapeworm would be growing within the peritoneal cavity (10 and 42 days). The 10‐ and 42‐day timepoints also correspond to timepoints used in previous experiments (Scharsack et al. 2007; Weber et al. 2017a, 2017b; Fuess et al. 2021). And (3) how this response might change within the timeframe of a breeding season, around 90 days. We used the 0–4 fibrosis scale described above. Two people (AKH and LEF) blind to treatment and population scored fibrosis (each fish was scored once). We also recorded fish mass, length, and sex. To the best of our ability, we spread treatments and timepoints across families within each population; sample sizes are provided in Table 1. Sample sizes are small for the 90‐day timepoint as this was added to take advantage of excess surviving fish. Throughout the experiment, there was a mortality rate of 11% (48 out of 418 fish), which did not appear to be driven by treatment. Mortality typically occurred days to weeks after injection.

**Table 1:** Sample sizes of fish that were scored for fibrosis across populations, timepoints, and treatments in our laboratory injection experiment. The number of families is indicated in parentheses

Population	Timepoint	PBS	Tapeworm	Alum	Tapeworm+Alum
Susceptible (Gosling) (14)	1 Day	11 (9)	10 (9)	10 (7)	10 (8)
	10 Days	10 (7)	10 (9)	10 (8)	10 (8)
	42 Days	11 (8)	10 (8)	10 (8)	10 (8)
	90 Days	3 (2)	2 (1)	8 (3)	4 (2)
Resistant (Roselle) (12)	1 Day	10 (8)	10 (7)	10 (8)	10 (7)
	10 Days	11 (8)	10 (7)	10 (8)	10 (7)
	42 Days	10 (7)	11 (7)	10 (7)	10 (8)
	90 Days	3 (2)	8 (1)	3 (1)	6 (1)
Ancestral (Sayward) (9)	1 Day	10 (6)	10 (6)	11 (5)	11 (7)
	10 Days	10 (7)	11 (7)	10 (6)	10 (5)
	42 Days	10 (5)	11 (5)	11 (5)	10 (6)
	90 Days	2 (1)	4 (1)	3 (2)	3 (1)

### ANALYSIS OF LABORATORY INJECTION EXPERIMENT

We built mixed effects models using *lmer* (Bates et al. 2015) and performed permutation tests using *predictmeans* (Luo et al. 2021). We first built models testing whether fibrosis score depends on the main effects of, and two‐ and three‐way interactions between, population, treatment, and time and assessed the importance of these interactions to model fit through permutations using the *permlmer* function from *predictmeans* (Luo et al. 2021). Given that the three‐way and two‐way interactions were significant and improved model fit, we next ran a series of smaller models on subsets of the data to test hypotheses concerning specific contrasts. These models tested: (1) Within a population, does fibrosis differ between treatments at a given timepoint? (2) Does the fibrosis response to a particular treatment vary between populations at a given timepoint? And (3) Within a population, does the fibrosis response to a given treatment change through time? For each of these questions, we looked at the main effect of treatment, population, or time and used permutation tests with 10,000 simulations using the *permmodels* function (Luo et al. 2021) to calculate parameter values and nonparametric permutation *P*‐values. If the main effect was significant, we then used pairwise contrasts (permuted *t*‐tests) to compare between groups using *predictmeans* (Luo et al. 2021). All models included room as a fixed effect. Family was included as a random effect, except for some models at 90 days where there was not enough variation. Denominator degrees of freedom for mixed models were estimated using *lmerTest* (Kuznetsova et al. 2017), which computes Kenward‐Roger approximations (Luke 2017). We ran all statistics using R version 4.0.3 (R Team 2021).

We explored several alternative analytical approaches. Given that fibrosis score is ordinal, we attempted to use cumulative link mixed models (clmms) using *Ordinal* (Christensen 2019). However, there was not enough variation in many of our comparisons for these models to run (complete separation). In cases where we could get clmms to run, we found very similar results. Even with our permutation approach, there were still some comparisons with little variation that generated an overfitted model. In these cases, we report results from a Kruskal‐Wallis test instead, or simply report the clear pattern. In our original data exploration, we found that fish size and sex did not influence fibrosis and that models were a better fit without them. We thus excluded these factors from subsequent analyses. This is consistent with laboratory findings that size and sex had no effect on fibrosis in response to tapeworm infections (Weber et al. 2021).

## Results

### FIELD SURVEY

From the 169 preserved fish collected from Roselle lake, we found six infected fish, giving an infection prevalence of 3.55%, which was lower than previous estimates for Roselle (7−40%) and Gosling lake (50–80%) (Weber et al. 2017b, 2021; De Lisle and Bolnick 2021).

**Figure 1:**
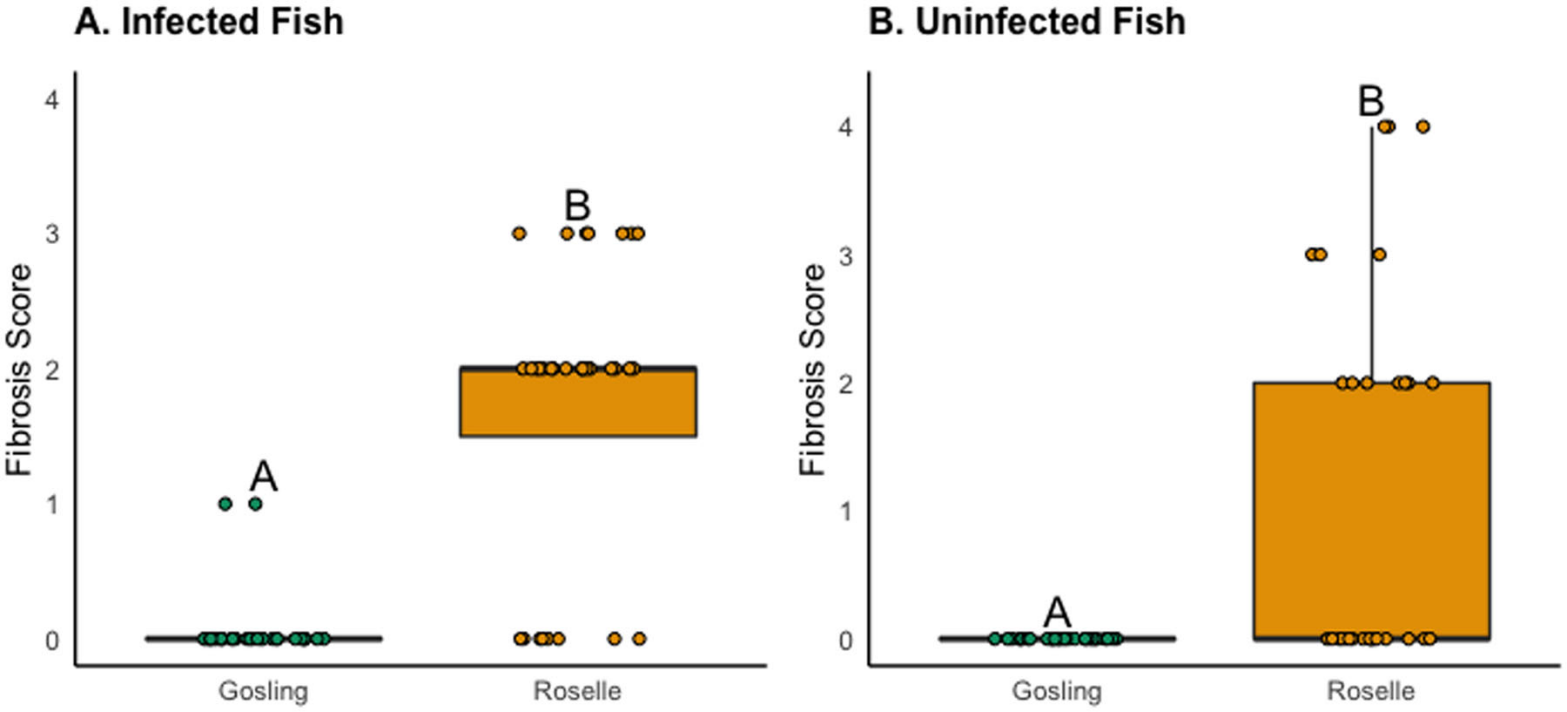
Fibrosis scores for *S. solidus* tapeworm infected (panel A) and currently uninfected (panel B) wild‐caught fish from Gosling and Roselle Lakes from 2018 field data. Raw data are represented as jittered points on the boxplot. Fish from Roselle had significantly more fibrosis in both the infected and uninfected groups relative to fish from Gosling.

Comparing the infected fish that we collected from each population, Gosling had a significantly higher infection intensity (GOS: mean = 3.87 tapeworms, SD = 5.75; RSL: mean = 2.31 tapeworms, SD = 1.82, GLM: *b*(Roselle) = −0.51, SE = 0.15, *P* < 0.001) and higher average tapeworm mass (GOS: mean = 52.61 mg, SD = 84.47; RSL: mean = 32.47 mg, SD = 55.03, *b*(Roselle) = 0.01, SE = 0.01, *P* = 0.27), although this was not statistically significant, likely because many infections fell below the 0.01 g limit of our field scale. When we compared the number of tapeworms above and below this threshold per lake, we found that Gosling had more large worms compared to Roselle (χ^2^ = 13.19, df = 1, *P* < 0.001). In infected fish, the degree of fibrosis was significantly higher in Roselle compared to Gosling (GOS: mean = 0.06, SD = 0.25; RSL: mean = 1.72, SD = 1.08; *W* = 132, *P* < 0.001; Fig. 1A). It is also of note that in seven of the fish from Roselle, we found small dead tapeworms encased in fibrosis, which we never observed in Gosling fish. When we compared uninfected fish across lakes, fibrosis scores were also significantly higher for Roselle (GOS: mean = 0, SD = 0; RSL: mean = 1.17, SD = 1.46; *W* = 263.5, *P* < 0.001; Fig. 1B). As we show below, the presence of fibrosis in “uninfected” Roselle fish may be a legacy of cleared infections. This supports other work demonstrating that across lakes (and in laboratory infection experiments), populations of stickleback with more fibrosis tend to have lower infection rates and smaller tapeworms, likely due to growth suppression. This has been confirmed for other resistant populations using laboratory infection experiments (Weber et al. 2021).

### LABORATORY INJECTION EXPERIMENT

**Figure 2:**
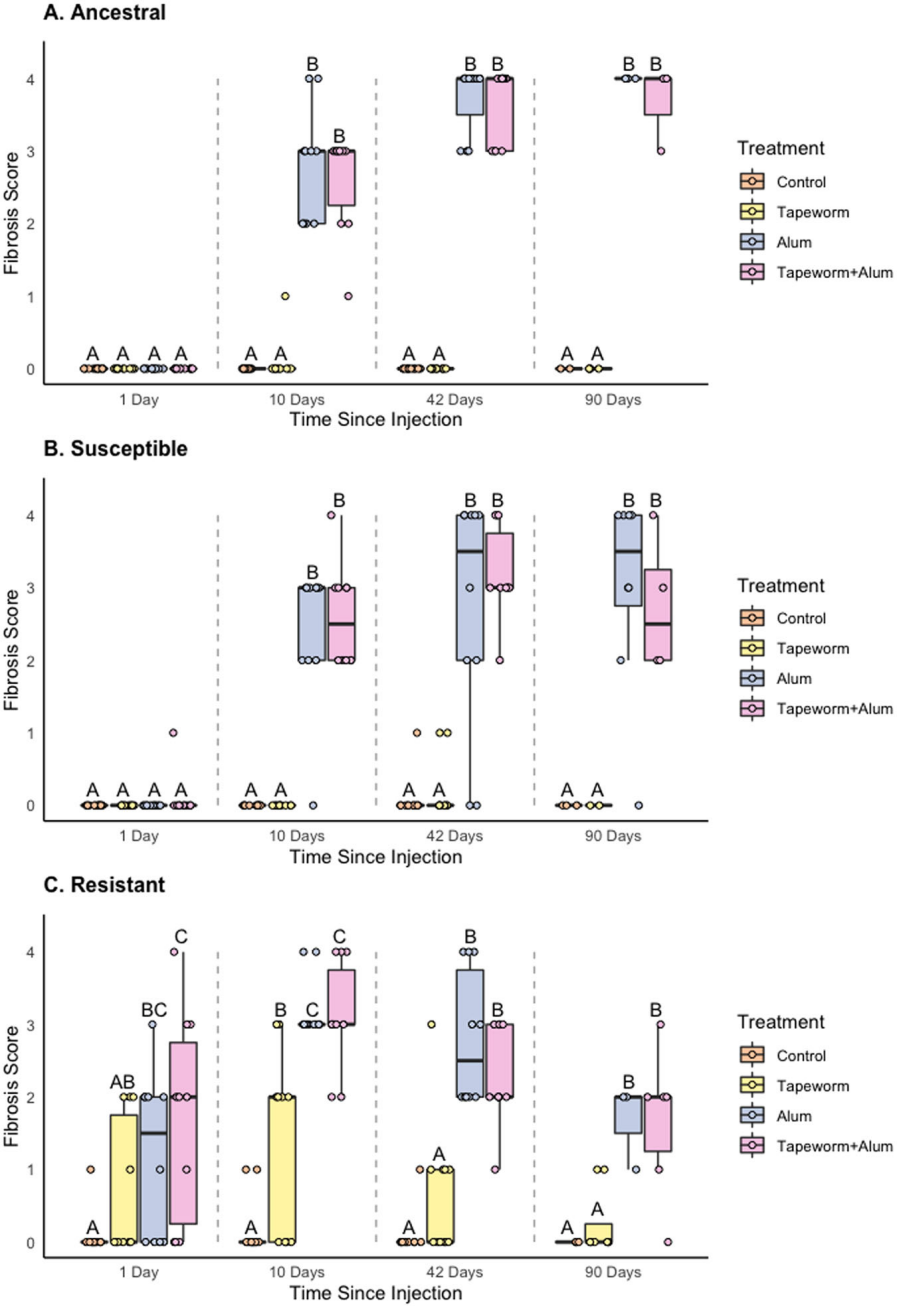
Fibrosis scores from the laboratory injection experiment for each injection treatment (Control, Tapeworm, Alum, and Tapeworm+Alum) at each timepoint (1, 10, 42, and 90 days point injection) for each population (Question 1 in the main text): (A) ancestral population, (B) susceptible population, and (C) resistant population. Letters denote significant differences between treatments within a timepoint (comparisons within gray dotted lines) using pairwise permutation *t*‐tests (*P*s < 0.05); raw data are shown as jittered points. All populations generated a strong fibrosis to the Alum and Tapeworm+Alum treatments by day 10. Roselle was the only population to respond within 24 hours and was also the only population to generate respond to the tapeworm treatment. Note that Figures 2, 3, 4 are replotted with the same data, each with a different focus and to illustrate results from different pairwise comparisons.

When examining the fibrosis response to injection, we found a significant three‐way interaction between treatment, timepoint, and population (*F*_6, 379_ = 5.10, *P* < 0.001). Once we broke this apart, all pairwise interactions were also significant (treatment*timepoint: *F*_3, 386_ = 16.49, *P* < 0.001; treatment*population: *F*_6, 391_ = 2.81, *P* = 0.01; timepoint*population: *F*_2, 200_ = 16.23, *P* < 0.001). The three‐way interaction model was a better fit relative to both the pairwise model and a model with no interactions (three‐way: ΔAIC = 0, pairwise: ΔAIC = 19.1, no interactions: ΔAIC = 89.6). To interpret these results, we used contrasts among subsets of data to address the three questions outlined in the methods. Figure 2 presents the full set of results, and subsequent figures focus attention on defined contrasts.

### QUESTION (1) WITHIN A POPULATION, DOES FIBROSIS DIFFER BETWEEN TREATMENTS AT A GIVEN TIMEPOINT?

For both the ancestral (Sayward) and the susceptible population (Gosling), fibrosis did not differ among treatments 1 day post injection, but did differ at 10, 42, and 90 days (Fig. 2A, B). For both populations, there was negligible fibrosis in the control and tapeworm treatments throughout and a strong fibrosis response to the alum and tapeworm+alum treatments starting at 10 days. The resistant population (Roselle) produced a different pattern, where fibrosis was significantly different between treatment groups at all four timepoints (Fig. 2C). The resistant population was the only one to show fibrosis at the first timepoint (to both alum treatments) and to develop fibrosis to the tapeworm treatment, which was lower than the response to both alum treatments. The model results are presented in Table 2, results of pairwise comparisons between treatments are summarized in Figure 2, and the least‐squared means and confidence intervals for these comparisons are reported in Table S1.

**Table 2:** Statistical results from mixed models and permutation tests (10,000 simulations) comparing fibrosis scores between treatments for a given timepoint for each population (Question 1). For comparisons marked with an asterisk, we had complete separation, or no response, so models would not run; when possible, we ran a Kruskal‐Wallis test instead. Results of pairwise comparisons between treatments are displayed in Figure 2A–C

Population	Comparison	Timepoint	Degrees of Freedom	*F* value	Permutation *P* value
Ancestral (Sayward)	Fibrosis ∼ Treatment	1 Day	*no response	*no response	*no response
		10 Days	36	78.86	<0.001
		42 Days	37	401.21	<0.001
		90 Days	8	177.00	0.002
Susceptible (Gosling)	Fibrosis ∼ Treatment	1 Day	33	1.32	0.07
		10 Days	30	75.45	<0.001
		42 Days	36	33.84	<0.001
		90 Days	12	9.33	0.004
Resistant (Roselle)	Fibrosis ∼ Treatment	1 Day	30	6.00	0.004
		10 Days	34	41.40	<0.001
		42 Days	34	30.36	<0.001
		90 Days	16	7.88	0.004

### QUESTION (2) DOES THE FIBROSIS RESPONSE TO A TREATMENT VARY BETWEEN POPULATIONS AT A GIVEN TIMEPOINT?

**Figure 3:**
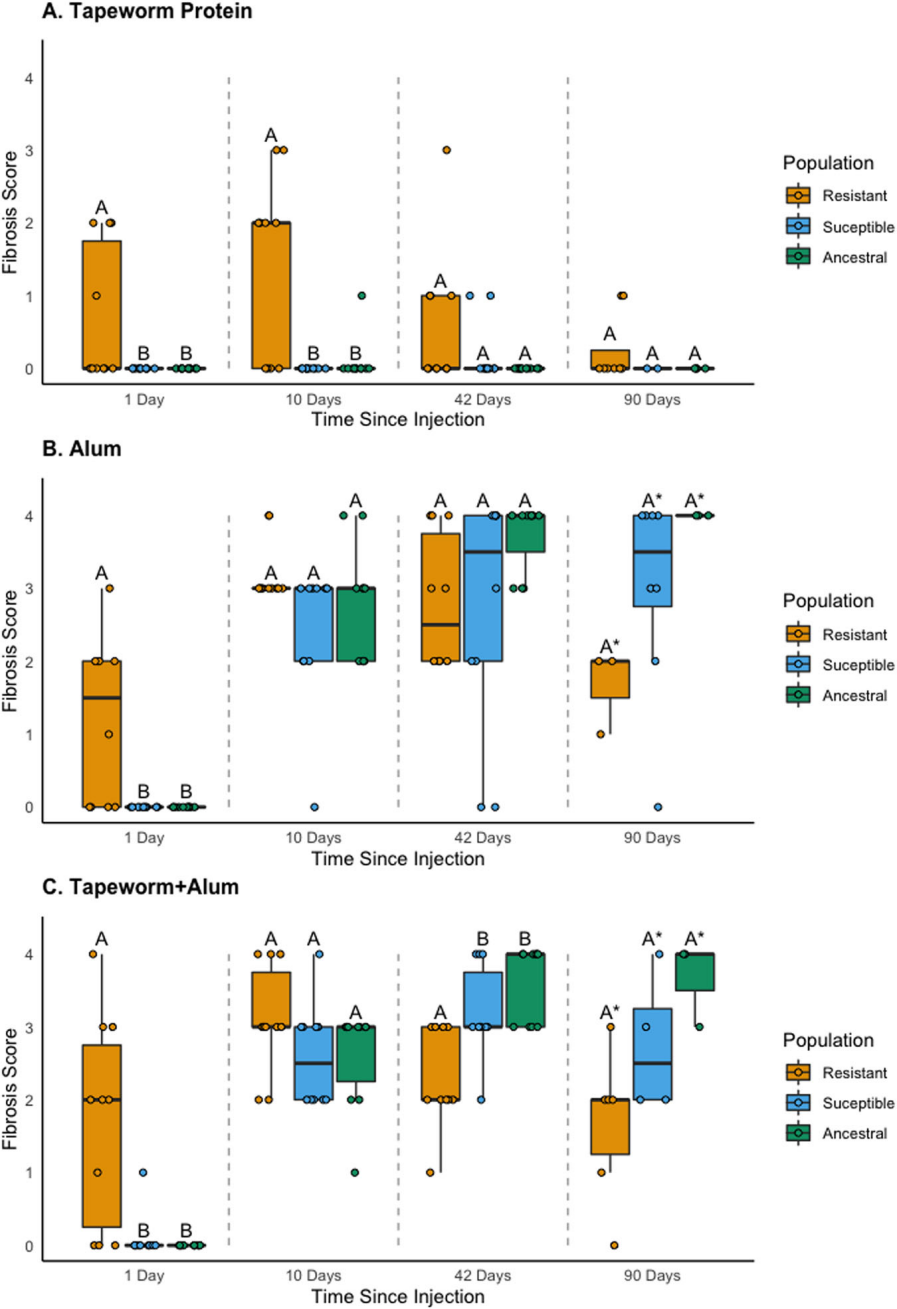
Fibrosis scores from the laboratory injection experiment for each population (Sayward, Gosling, and Roselle) at each timepoint (1, 10, 42, and 90 days post injection) for each injection treatment (Question 2 in the main text): (A) tapeworm protein, (B) alum, and (C) tapeworm + alum. The control treatment (PBS) is not pictured as there was little response in any of the populations. Letters denote significant differences between populations within a timepoint (comparisons within gray dotted lines) using pairwise permutation *t*‐tests (*P*s < 0.05); raw data are shown as jittered points. An asterisk denotes reduced statistical power due to small sample sizes at the 90‐day timepoint. The responses from the susceptible and ancestral populations were indistinguishable, whereas the resistant population followed a different pattern in all three treatments. Note that Figures 2, 3, 4 are replotted with the same data, each with a different focus and to illustrate results from different pairwise comparisons.

For the control treatment, there was little fibrosis and populations did not differ at any timepoint. For the tapeworm treatment, populations differed in their fibrosis response at both 1 and 10 days, but not at 42 and 90 days, with the resistant population being the only one to produce fibrosis to this treatment (Fig. 3A). For the alum treatment, fibrosis was significantly different between populations for the first timepoint only (Fig. 3B). Specifically, the resistant population was the only one to produce fibrosis within 24 hours, but the other populations caught up to produce similar levels of fibrosis for the middle timepoints. At 90 days, there was a trend for the alum treatment, with a decreasing response for the resistant population, although small sample sizes limited our statistical power. For the tapeworm+alum treatment, fibrosis was significantly different between populations for 1 and 42 days, with the resistant population being the only one to respond on day 1 with a decreased response at 42 days relative to the other two populations (Fig. 3C). Again, for 90 days there was a decreasing response for the resistant population, although small sample sizes limited our statistical power. The model results are presented in Table 3, and results of pairwise comparisons are summarized in Figure 3.

**Table 3:** Statistical results from mixed models and permutation tests (10,000 simulations) comparing fibrosis scores between populations for a given timepoint and treatment (Question 2). For comparisons marked with an asterisk we had complete separation, or no response, so models would not run; when possible, we ran a Kruskal‐Wallis test instead. Pairwise comparisons between populations are presented in Figure 2D–F

Treatment	Comparison	Timepoint	Degrees of Freedom	*F* value	Permutation *P* value
Control (PBS)	Fibrosis ∼ Population	1 Day	*no response df = 2	*no response χ ^2^ = 2.1	*no response P = 0.35
		10 Days	27	1.71	0.31
		42 Days	27	0.03	0.98
		90 Days	*no response	*no response	*no response
Tapeworm protein	Fibrosis ∼ Population	1 Day	20	3.63	0.042
		10 Days	27	11.60	0.001
		42 Days	16	1.75	0.24
		90 Days	11	0.79	0.66
Alum	Fibrosis ∼ Population	1 Day	*only Roselle df = 2	*only Roselle χ ^2^ = 14.96	*only Roselle P < 0.001
		10 Days	16	3.37	0.10
		42 Days	16	1.95	0.22
		90 Days	10	3.81	0.11
Tapeworm + Alum	Fibrosis ∼ Population	1 Day	15	10.71	0.008
		10 Days	26	1.34	0.32
		42 Days	16	11.07	0.005
		90 Days	2	0.99	0.47

### QUESTION (3) WITHIN A POPULATION, DOES THE FIBROSIS RESPONSE TO A GIVEN TREATMENT CHANGE THROUGH TIME?

**Figure 4:**
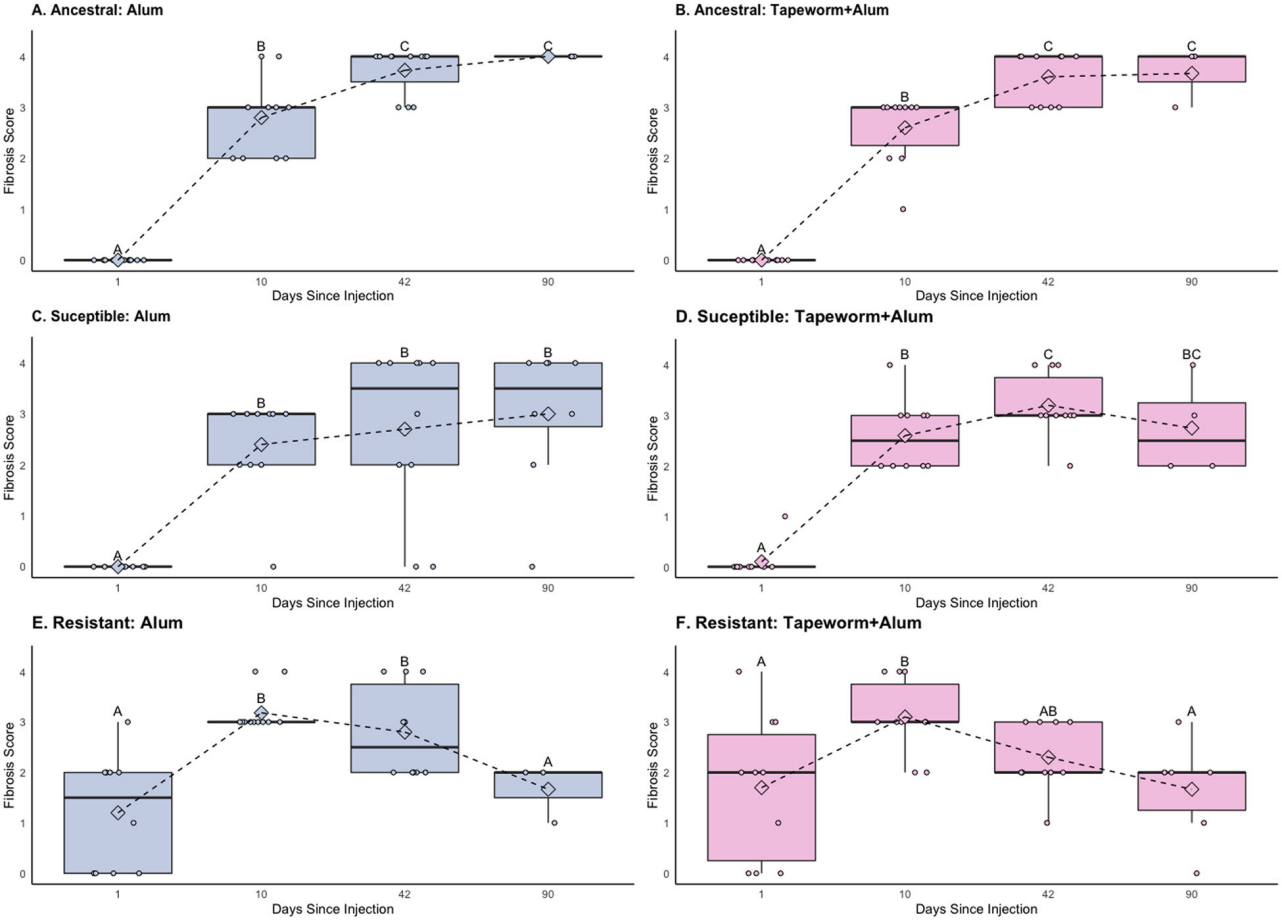
Fibrosis scores from alum and tapeworm + alum injection treatments (control and tapeworm treatments not pictured) through time (1, 10, 41, and 90 days post injection) for each population (Question 3 in the main text): (A) ancestral population, alum treatment; (B) ancestral population, tapeworm + alum treatment; (C) susceptible population, alum treatment; (D) susceptible population, tapeworm + alum treatment; (E) resistant population, alum treatment; and (F) resistant population, tapeworm + alum treatment. Letters denote significant differences between timepoints using pairwise permutation *t*‐tests (*P*s < 0.05), raw data are shown as jittered points. The resistant population initiated fibrosis earlier, within 24 hours, and then showed evidence of a decreased response (attenuation) in the last timepoint(s) of the experiment, unlike the susceptible or ancestral populations, both of which either increased or maintained a high level of fibrosis after day 10. Note that Figures 2, 3, 4 are replotted with the same data, each with a different focus and to illustrate results from different pairwise comparisons.

For both the ancestral and susceptible populations, fibrosis to the control and tapeworm treatments did not differ through time (negligible fibrosis at all timepoints); however, fibrosis did change through time for both the alum and the tapeworm+alum treatments (Fig. 4A–D). For both populations, there was little response on day 1, an increase in fibrosis from 10 to 42 days, and a continuation of high fibrosis at 90 days, showing little evidence of attenuation, except for a slight decrease from 42 to 90 days for the susceptible population in the tapeworm+alum treatment. For the resistant population, there was again no significant effect of time for the control and tapeworm treatments, although we note for the tapeworm treatment that there was a trend where fibrosis appeared to peak at 10 days and then decrease at 42 and 90 days. Fibrosis did significantly change through time for the other treatments, where fibrosis was detected at the first timepoint, peaked at 10 days, and by 90 days had decreased, showing clear attenuation of the response (Fig. 4E, F). The model results are presented in Table 4, and results of pairwise comparisons between populations are summarized in Figure 4.

**Table 4:** Statistical results from mixed models and permutation tests (10,000 simulations) comparing fibrosis scores for a given treatment through time for each population (Question 3). For comparisons marked with an asterisk, we had complete separation, or no response, so models would not run; when possible, we ran a Kruskal‐Wallis test instead. Pairwise comparisons between timepoints are displayed in Figure 2G–L

Population	Comparison	Treatment	Degrees of Freedom	*F* value	Permutation *P* value
Ancestral (Sayward)	Fibrosis ∼ Timepoint	Control	*no response	*no response	*no response
		Tapeworm	29	0.71	0.74
		Alum	29	178.24	<0.001
		Tapeworm + Alum	29	106.30	0.001
Susceptible (Gosling)	Fibrosis ∼ Timepoint	Control	30	0.78	0.52
		Tapeworm	27	1.41	0.30
		Alum	21	13.80	<0.001
		Tapeworm + Alum	25	55.47	<0.001
Resistant (Roselle)	Fibrosis ∼ Timepoint	Control	29	0.26	0.93
		Tapeworm	29	2.07	0.13
		Alum	27	12.24	<0.001
		Tapeworm + Alum	31	4.45	0.02

## Discussion

Striking differences in parasite resistance between related groups, even to the same parasite, are common in nature, and yet, we often lack an understanding of the immunological mechanisms that generate this important variation. Although many evolutionary studies emphasize the broad outcomes of infection (e.g., success/failure or parasite load), we instead take a stepwise approach that breaks up different components of the host response through time to better understand resistance evolution in populations of threespine stickleback. We compared an ancestral marine population and two lake populations that differ in their resistance to a freshwater tapeworm. Using naïve fish raised in a common environment and a novel immune challenge assay, we set out to test if (H1) variation in a resistance phenotype (fibrosis) observed in nature was being driven only by the environment, (H2) variation was due to population‐level differences in the ability to detect and initiate a respond to tapeworm antigens, or (H3) variation was due to population‐level differences in the ability to actuate a strong fibrosis response in the peritoneal cavity. We also tested for variation in the rate of initiation and resolution of fibrosis, with the prediction that selection in the resistant population may have favored rapid initiation (to control still‐small tapeworms) and quicker resolution (to mitigate long‐term costs of fibrotic pathology) (De Lisle and Bolnick 2021; Weber et al. 2021).

We found that populations differed in their response to the injected immune challenges despite being raised in a common environment. This suggests that variation in resistance phenotypes across populations is heritable and thus evolved, rather than being due solely to environmental effects (rejecting H1). We also rejected H3, because, given enough time, all populations produced a robust fibrosis response in their peritoneal cavity to a general immune adjuvant (alum). This result is consistent with a follow‐up study that used our alum injection protocol on a phylogenetically diverse sample of 17 fishes, showing a deep ancestral ability to actuate peritoneal fibrosis (Vrtílek and Bolnick 2021). Instead, we found that our data support H2: only the resistant population exhibited a fibrosis response to the tapeworm antigen alone, suggesting that population‐level differences in resistance reflect variation in the earliest stages of immune activation. As predicted, the resistant population also differed in the timing of fibrosis. Although all three populations actuated fibrosis in response to alum, the resistant population produced fibrosis faster, within 24 hours, and then attenuated fibrosis at later timepoints, which was not the case for the other populations. From these results, we conclude that the capacity to actuate peritoneal fibrosis is an ancient trait conserved by all three genotypes, but the propensity to initiate fibrosis in response to tapeworm antigens is recently evolved, following the colonization of freshwater lakes by marine stickleback (judging by the modern marine sample). Parasite resistance in certain freshwater populations appears to be driven by the ability of hosts to recognize and rapidly initiate a respond to *S. solidus* infections.

The immunological and genetic mechanisms underlying fibrosis evolution are unclear at present, but we can draw some strong preliminary inferences. Our observation that all three populations were capable of actuating strong peritoneal fibrosis indicates that core fibroblast activation pathways to produce collagen and fibronectin are intact. The difference must therefore proceed the actual production of fibrosis, either involving parasite recognition or response initiation. These are notoriously difficult to separate, but we present two lines of evidence that the difference entails initiation rather than recognition. First, the gene family most commonly associated with macroparasite recognition, including helminths, is the Major Histocompatibility Complex IIβ (MHC IIβ) (Kurtz et al. 2004; Eizaguirre et al. 2010; Radwan et al. 2020), but two studies found no MHC IIβ alleles associated with *S. solidus* prevalence (Stutz and Bolnick 2017; Peng et al. 2021). Experimental infections of lab‐reared hybrids between Gosling Lake and another resistant population (Roberts lake) revealed QTL for fibrosis and tapeworm growth, but no QTL contained known pattern‐recognition proteins such as MHC (Weber et al. 2021). Then, there is the consideration that adaptive immune responses (of which MHC IIβ is a part) typically take many days to initiate (especially in naïve fish that lack prior immune memory, and in ectotherms in cold water) (Wegner et al. 2007). In contrast, we see the start of a fibrotic response to tapeworm protein within 24 hours for the resistant population. Second, transcriptomic data reveal that susceptible and resistant populations exhibit mostly similar gene expression changes in response to *S. solidus* infection (Lohman et al. 2017; Fuess et al. 2021), with few genotype by infection interactions. These shared responses suggest that the susceptible population is recognizing *S. solidus* infections. These observations lead us to infer that evolved population differences in resistance likely entail activation switches that act after initial parasite recognition.

At first glance, the most parsimonious evolutionary model to explain our results is that the colonizing marine fish gained an anti‐tapeworm fibrosis response in some lakes, but not others. Gosling fish (susceptible population) resemble the ancestor‐like marine outgroup in that they permit rapid tapeworm growth (Weber et al. 2017a) and lack fibrosis in the wild and in the lab (after either infection or injection with tapeworm antigens). However, it is also possible that freshwater colonists typically evolve fibrosis in response to *S. solidus* infections, but that the costs of fibrosis (De Lisle and Bolnick 2021; Weber et al. 2021) favored secondary loss of fibrosis with infection in some populations. This loss would constitute a tolerance strategy, resulting in renewed susceptibility to fast‐growing tapeworms. This less‐parsimonious hypothesis is supported by other recent studies that provide evidence for strong selection in Gosling Lake favoring fixation of several partial gene deletions (Weber et al. 2021), and upregulation of a fibrosis‐suppression pathway (Fuess et al. 2021).

Our results also suggest that within the resistant population, there has been selection on the timing of both initiating and resolving the fibrosis response. Biologically, this makes sense because if fish can respond early, when tapeworms are small, they are likely to be more successful in clearing the infection. This could explain what we witnessed in the field, where small dead tapeworms were encased in fibrosis. Even if fibrosis does not successfully kill tapeworms, early initiation could still limit their growth, allowing fish to avoid many of the negative consequences imposed by large tapeworms. Interestingly, fibrosis and other injury repair pathways have also been implicated in helminth suppression in mice and humans (Allen and Sutherland 2014). If fish can successfully clear infection, resolving the fibrosis response is likely adaptative as we known that fibrosis negatively impacts swimming ability (Matthews et al. unpubl. ms.) and reproduction (De Lisle and Bolnick 2021; Weber et al. 2021). It is clear from work in other systems that variation in the timing, and not necessarily the magnitude, of the immune response can lead to striking differences in infection outcomes (Duneau et al. 2017). In all three populations, however, significant fibrosis persisted to 90 days after a single immune challenge (alum treatments). This highlights the potential long‐term cost of mounting such a response, particularly in a short‐lived fish, and supports the idea that susceptible lake populations may be using a tolerance strategy for dealing with *S. solidus*. It also explains why we observe fibrotic individuals without *S. solidus* in the wild for Roselle.

Our results are in line with other studies in stickleback that have found consistent population‐level variation in *S. solidus* susceptibility and growth suppression, which remains when fish are experimentally infected in the lab and reciprocally exposed to tapeworms from different populations (Kalbe et al. 2016; Weber et al. 2017b; Piecyk et al. 2019b). It is worth noting that tapeworm populations also consistently vary in virulence, for which size is a good proxy (Kalbe et al. 2016; Ritter et al. 2017). Although few studies have examined fibrosis as a response to tapeworms, our work does fit into other immunological studies that examine the timing of the host response. This work suggests that initial recognition of the parasite is followed by early mobilization of immune cells (within 7 days post exposure) and that *S. solidus* can only be successfully cleared during the early phase of the infection when the tapeworm is small (Hammerschmidt and Kurtz 2007; Scharsack et al. 2007; Barber and Scharsack 2010). Following this early period, rapid parasite growth, active immune suppression, and changing of surface antigens allow the tapeworm to evade subsequent fish immunity (Scharsack et al. 2004, 2013; Hammerschmidt and Kurtz 2005), and antibody‐mediated adaptive immunity is not found to be upregulated with tapeworm infections, even at later stages (Scharsack et al. 2007; Barber and Scharsack 2010). This suggests that early detection and initiation of rapid innate responses that clear infection or suppress tapeworm growth are key characteristics of host resistance, whereas susceptible or tolerant populations likely react more slowly, if at all, and allow tapeworms to grow to a large size, which is required for successful reproduction in the definitive host (Barber et al. 2004).

Our study worked to isolate the genetic contribution to variation in the response to infection across populations, by raising all fish in the same laboratory conditions and injecting them with different immune challenges (removing, e.g., variation in exposure or resources). This approach can be informative, as it allows for the isolation of genes from ecology, but translating our results back to wild populations must be done with some caution. It is clear that ecology can interact with genetics to play an important role in shaping host responses to infection and generating variation between populations (Hawley and Altizer 2011; Leung et al. 2018). Additionally, using the same tapeworm extract allows us to easily compare across populations, but does not address how a live tapeworm might interfere with the fibrosis response (Steinel and Bolnick 2018; Piecyk et al. 2019a; Fuess et al. 2021), and beyond that, how variation in the tapeworms themselves might further shape host responses (e.g., local adaptation, gene‐for‐gene epistasis between species). We also acknowledge that our study is limited to only three populations, and it will be informative to determine if the same patterns exist between other susceptible and resistant lake populations. Ongoing gene expression work using samples collected from this experiment will also provide more insights into the genetic mechanisms and immunological pathways underlying these patterns and will give more detail about other aspects of the immune response beyond the visual fibrosis score used here.

By taking a stepwise approach to isolate different stages of the host response to infection, we were able to uncover clear evidence that differences arising at key stages of the immune response are likely driving variation in parasite resistance between closely related host populations. Applying this approach, of partitioning variance across infection stages, in a variety of wild systems is likely to provide a more nuanced understanding of the mechanisms generating variation in parasite resistance and insight into host‐parasite coevolution.

## AUTHOR CONTRIBUTIONS

AKH, KCS, and DIB carried out all field work. KCS dissected preserved fish from Roselle. AKH, LEF, and DIB designed the laboratory experiment. AKH, LEF, MLK, MFM, and JMM carried out the laboratory experiment. AKH performed all analyses, made all figures, and wrote the manuscript. All authors provided feedback on the manuscript.

## DATA ARCHIVING

All data and code associated with this manuscript can be found on Dryad, https://doi.org/10.5061/dryad.0cfxpnw45.

Associate Editor: Dr. Katrina Lythgoe

## Supporting information


**Figure S1**: Map of sampling locations for each of the study populations on Vancouver Island, Canada. Map curtesy of Vivid Maps 2021.
**Table S1**: Least squared means and confidence intervals (from permuted t‐tests) for fibrosis response results from the laboratory injection experiment.

**SI**: Video.

## References

[evl3274-bib-0001] Allen, J. E. , and T. E. Sutherland 2014. Host protective roles of type 2 immunity: parasite killing and tissue repair, flip sides of the same coin. Semin. Immunol. 26:329–340. 25028340 10.1016/j.smim.2014.06.003 PMC4179909

[evl3274-bib-0002] Armour, E. , T. L. Bruner , J. K. Hines , and M. W. Butler 2020. Low‐dose immune challenges result in detectable levels of oxidative damage. J. Exp. Biol. 223:jeb220095. 32054680 10.1242/jeb.220095

[evl3274-bib-0003] Barber, I. , and J. P. Scharsack 2010. The three‐spined stickleback‐*Schistocephalus solidus* system: an experimental model for investigating host‐parasite interactions in fish. Parasitology 137:411–424. 19835650 10.1017/S0031182009991466

[evl3274-bib-0004] Barber, I. , and P. A. Svensson 2003. Effects of experimental *Schistocephalus solidus* infections on growth, morphology and sexual development of female three‐spined sticklebacks, *Gasterosteus aculeatus* . Parasitology 126:359–367. 12741515 10.1017/s0031182002002925

[evl3274-bib-0005] Barber, I. , P. Walker , and P. A. Svensson 2004. Behavioural responses to simulated avian predation in female three spined sticklebacks: the effect of experimental *Schistocephalus solidus* infections. Behaviour 141:1425–1440.

[evl3274-bib-0006] Barron, D. G. , S. S. Gervasi , J. N. Pruitt , and L. B. Martin 2015. Behavioral competence: how host behaviors can interact to influence parasite transmission risk. Curr. Opin. Behav. Sci. 6:35–40.

[evl3274-bib-0007] Bates, D. , M. Maechler , B. Bolker , and S. Walker 2015. Fitting linear mixed‐effects models using lme4. J. Stat. Softw. 67:1–48.

[evl3274-bib-0008] Bell, M. A. , and S. A. Foster 1994. The evolutionary biology of the threespine stickleback (Oxford science publications). Oxford Univ. Press, Oxford, U.K.

[evl3274-bib-0009] Blake, R. W. , P. Y. L. Kwok , and K. H. S. Chan 2006. Effects of two parasites, *Schistocephalus solidus* (Cestoda) and *Bunodera* spp. (Trematoda), on the escape fast‐start performance of three‐spined sticklebacks. J. Fish Biol. 69:1345–1355.

[evl3274-bib-0010] Boots, M. , A. Best , M. R. Miller , and A. White 2009. The role of ecological feedbacks in the evolution of host defence: what does theory tell us? Philos. Trans. R. Soc. B Biol. Sci. 364:27–36. 10.1098/rstb.2008.0160 PMC2666696 18930880

[evl3274-bib-0011] Caldera, E. J. , and D. I. Bolnick 2008. Effects of colonization history and landscape structure on genetic variation within and among threespine stickleback (*Gasterosteus aculeatus*) populations in a single watershed. Evol. Ecol. Res. 10:575–598.

[evl3274-bib-0012] Christensen, R. H. B. 2019. ordinal: regression models for ordinal data. R package.

[evl3274-bib-0013] Coakley, G. , A. H. Buck , and R. M. Maizels 2016. Host parasite communications—Messages from helminths for the immune system: parasite communication and cell‐cell interactions. Mol. Biochem. Parasitol. 208:33–40. 27297184 10.1016/j.molbiopara.2016.06.003 PMC5008435

[evl3274-bib-0014] Divino, J. , and E. Schultz 2014. Juvenile threespine stickleback husbandry: standard operating procedures of the Schultz lab. University of Connecticut, Storrs, CT.

[evl3274-bib-0015] Duneau, D. , J. B. Ferdy , J. Revah , H. Kondolf , G. A. Ortiz , B. P. Lazzaro , et al. 2017. Stochastic variation in the initial phase of bacterial infection predicts the probability of survival in *D. melanogaster* . Elife 6:e28298. 29022878 10.7554/eLife.28298 PMC5703640

[evl3274-bib-0016] Eizaguirre, C. , T. L. Lenz , R. D. Sommerfeld , C. Harrod , M. Kalbe , and M. Milinski 2010. Parasite diversity, patterns of MHC II variation and olfactory based mate choice in diverging three‐spined stickleback ecotypes. Evol. Ecol. 25:605–622.

[evl3274-bib-0017] Fernandes, L. D. , P. Lemos‐Costa , P. R. Guimarães , J. N. Thompson , and M. A. M. de Aguiar 2019. Coevolution creates complex mosaics across large landscapes. Am. Nat. 194:217–229. 31318284 10.1086/704157

[evl3274-bib-0018] Fuess, L. E. , J. N. Weber , S. den Haan , N. C. Steinel , K. C. Shim , and D. I. Bolnick 2021. Between‐population differences in constitutive and infection‐induced gene expression in threespine stickleback. Mol. Ecol. 30:6791‐6805. 34582586 10.1111/mec.16197 PMC8796319

[evl3274-bib-0019] Ganeshan, K. , and A. Chawla 2014. Metabolic regulation of immune responses. Annu. Rev. Immunol. 32:609–634. 24655299 10.1146/annurev-immunol-032713-120236 PMC5800786

[evl3274-bib-0020] Giles, N. 1983. Behavioural effects of the parasite *Schistocephalus solidus* (Cestoda) on an intermediate host, the three‐spined stickleback, *Gasterosteus aculeatus* L. Anim. Behav. 31:1192–1194.

[evl3274-bib-0021] Hall, M. D. , G. Bento , and D. Ebert 2017. The evolutionary consequences of stepwise infection processes. Trends Ecol. Evol. 32:612–623. 28648806 10.1016/j.tree.2017.05.009

[evl3274-bib-0022] Hall, M. D. , J. Routtu , and D. Ebert 2019. Dissecting the genetic architecture of a stepwise infection process. Mol. Ecol. 28:3942–3957. 31283079 10.1111/mec.15166

[evl3274-bib-0023] Hammerschmidt, K. , and J. Kurtz 2005. Surface carbohydrate composition of a tapeworm in its consecutive intermediate hosts: individual variation and fitness consequences. Int. J. Parasitol. 35:1499–1507. 16198355 10.1016/j.ijpara.2005.08.011

[evl3274-bib-0024] Hammerschmidt, K. , and J. Kurtz 2007. *Schistocephalus solidus*: establishment of tapeworms in sticklebacks ‐ fast food or fast lane? Exp. Parasitol. 116:142–149. 17296178 10.1016/j.exppara.2006.12.013

[evl3274-bib-0025] Hawley, D. M. , and S. M. Altizer 2011. Disease ecology meets ecological immunology: understanding the links between organismal immunity and infection dynamics in natural populations. Funct. Ecol. 25:48–60.

[evl3274-bib-0026] Henrich, T. , N. Hafer , and K. B. Mobley 2014. Effects of VIE tagging and partial tissue sampling on the immune response of three‐spined stickleback *Gasterosteus aculeatus* . J. Fish Biol. 85:965–971. 25060133 10.1111/jfb.12471

[evl3274-bib-0027] Kalbe, M. , C. Eizaguirre , J. Scharsack , and P. J. Jakobsen 2016. Reciprocal cross infection of sticklebacks with the diphyllobothriidean cestode *Schistocephalus solidus* reveals consistent population differences in parasite growth and host resistance. Parasit. Vectors 9:1–12. 26951744 10.1186/s13071-016-1419-3 PMC4782366

[evl3274-bib-0028] Karvonen, A. , and O. Seehausen 2012. The role of parasitism in adaptive radiations‐ when might parasites promote and when might they constrain ecological speciation? Int. J. Ecol. 2012:1–20.

[evl3274-bib-0029] Khan, I. , D. Agashe , and J. Rolff 2017. Early‐life inflammation, immune response and ageing. Proc. R. Soc. B Biol. Sci. 284:20170125. 10.1098/rspb.2017.0125 PMC5360934 28275145

[evl3274-bib-0030] Kirch, M. , A. Romundset , M. T. P. Gilbert , F. C. Jones , and A. D. Foote 2021. Ancient and modern stickleback genomes reveal the demographic constraints on adaptation. Curr. Biol. 31:2027–2036.e8. 33705715 10.1016/j.cub.2021.02.027

[evl3274-bib-0031] Kool, M. , K. Fierens , and B. N. Lambrecht 2012. Alum adjuvant: some of the tricks of the oldest adjuvant. J. Med. Microbiol. 61:927–934. 22174375 10.1099/jmm.0.038943-0

[evl3274-bib-0032] Kurtz, J. , M. Kalbe , P. B. Aeschlimann , M. A. Häberli , K. M. Wegner , T. B. H. H. Reusch , et al. 2004. Major histocompatibility complex diversity influences parasite resistance and innate immunity in sticklebacks. Proc. R. Soc. B Biol. Sci. 271:197–204. 10.1098/rspb.2003.2567 PMC1691569 15058398

[evl3274-bib-0033] Kuznetsova, A. , P. B. Brockhoff , and R. H. B. Christensen 2017. lmerTest package: tests in linear mixed effects models. J. Stat. Softw. 82:1–26.

[evl3274-bib-0034] Leung, J. M. , S. A. Budischak , H. C. The , C. Hansen , R. Bowcutt , R. Neill , et al. 2018. Rapid environmental effects on gut nematode susceptibility in rewilded mice. PLoS Biol. 16:1–28. 10.1371/journal.pbio.2004108 PMC5843147 29518091

[evl3274-bib-0035] De Lisle, S. P. , and D. I. Bolnick 2021. Male and female reproductive fitness costs of an immune response in natural populations. Evolution 75:2509–2523. 33991339 10.1111/evo.14266 PMC8488946

[evl3274-bib-0036] Lohman, B. K. , N. C. Steinel , J. N. Weber , and D. I. Bolnick 2017. Gene expression contributes to the recent evolution of host resistance in a model host parasite system. Front. Immunol. 8:1071. 28955327 10.3389/fimmu.2017.01071 PMC5600903

[evl3274-bib-0037] Luke, S. G. 2017. Evaluating significance in linear mixed‐effects models in R. Behav. Res. Methods 49:1494–1502. 27620283 10.3758/s13428-016-0809-y

[evl3274-bib-0038] Luo, D. , S. Ganesh , and J. Koolaard 2021. Predictmeans: calculate predicted means for linear models.

[evl3274-bib-0039] MacNab, V. , I. Katsiadaki , and I. Barber 2009. Reproductive potential of *Schistocephalus solidus*‐infected male three‐spined stickleback *Gasterosteus aculeatus* from two U.K. populations. J. Fish Biol. 75:2095–2107. 20738675 10.1111/j.1095-8649.2009.02411.x

[evl3274-bib-0040] Maizels, R. M. , H. H. Smits , and H. J. McSorley 2018. Modulation of host immunity by helminths: the expanding repertoire of parasite effector molecules. Immunity 49:801–818. 30462997 10.1016/j.immuni.2018.10.016 PMC6269126

[evl3274-bib-0041] Milinski, M. 1985. Risk of predation of parasitized sticklebacks (*Gasterosteus Aculeatus L*.) under competition for food. Behaviour 93:203–216.

[evl3274-bib-0042] Motran, C. C. , L. Silvane , L. S. Chiapello , M. G. Theumer , L. F. Ambrosio , X. Volpini , et al. 2018. Helminth infections: recognition and modulation of the immune response by innate immune cells. Front. Immunol. 9:1–12. 29670630 10.3389/fimmu.2018.00664 PMC5893867

[evl3274-bib-0043] Orr, T. S. C. , C. A. Hopkins , and G. H. Charles 1969. Host specificity and rejection of *Schistocephalus solidus* . Parasitology 59:683–690.

[evl3274-bib-0044] Papkou, A. , C. S. Gokhale , A. Traulsen , and H. Schulenburg 2016. Host–parasite coevolution: why changing population size matters. Zoology 119:330–338. 27161157 10.1016/j.zool.2016.02.001

[evl3274-bib-0045] Peng, F. , K. M. Ballare , S. Hollis Woodard , S. den Haan , and D. I. Bolnick 2021. What evolutionary processes maintain MHC IIB diversity within and among populations of stickleback? Mol. Ecol. 30:1659–1671. 33576071 10.1111/mec.15840 PMC8049082

[evl3274-bib-0046] Piecyk, A. , M. Ritter , and M. Kalbe 2019a. The right response at the right time: exploring helminth immune modulation in sticklebacks by experimental coinfection. Mol. Ecol. 28:2668–2680. 30993799 10.1111/mec.15106 PMC6852435

[evl3274-bib-0047] Piecyk, A. , O. Roth , and M. Kalbe 2019b. Specificity of resistance and geographic patterns of virulence in a vertebrate host‐parasite system. BMC Evol. Biol. 19:1–14. 30890121 10.1186/s12862-019-1406-3 PMC6425677

[evl3274-bib-0048] Radwan, J. , W. Babik , J. Kaufman , T. L. Lenz , and J. Winternitz 2020. Advances in the evolutionary understanding of MHC polymorphism. Trends Genet. 36:298–311. 32044115 10.1016/j.tig.2020.01.008

[evl3274-bib-0049] Ritter, M. , M. Kalbe , and T. Henrich 2017. Virulence in the three‐spined stickleback specific parasite *Schistocephalus solidus* is inherited additively. Exp. Parasitol. 180:133–140. 28242354 10.1016/j.exppara.2017.02.016

[evl3274-bib-0050] Roberts Kingman, G. A. , D. N. Vyas , F. C. Jones , S. D. Brady , H. I. Chen , K. Reid , et al. 2021. Predicting future from past: the genomic basis of recurrent and rapid stickleback evolution. Sci. Adv. 7:eabg5285. 34144992 10.1126/sciadv.abg5285 PMC8213234

[evl3274-bib-0051] Roy, B. A. , and J. W. Kirchner 2000. Evolutionary dynamics of pathogen resistance and tolerance. Evolution 54:51–63. 10937183 10.1111/j.0014-3820.2000.tb00007.x

[evl3274-bib-0072] R Core Team . 2021. R: A language and environment for statistical computing. R Foundation for Statistical Computing, Vienna, Austria. https://www.R-project.org/

[evl3274-bib-0052] Scharsack, J. P. , M. Kalbe , R. Derner , J. Kurtz , and M. Milinski 2004. Modulation of granulocyte responses in three‐spined sticklebacks *Gasterosteus aculeatus* infected with the tapeworm *Schistocephalus solidus* . Dis. Aquat. Organ. 59:141–150. 15212281 10.3354/dao059141

[evl3274-bib-0053] Scharsack, J. P. , K. Koch , and K. Hammerschmidt 2007. Who is in control of the stickleback immune system: interactions between *Schistocephalus solidus* and its specific vertebrate host. Proc. R. Soc. B Biol. Sci. 274:3151–3158. 10.1098/rspb.2007.1148 PMC2293945 17939987

[evl3274-bib-0054] Scharsack, J. P. , A. Gossens , F. Franke , and J. Kurtz 2013. Excretory products of the cestode, Schistocephalus solidus, modulate invitro responses of leukocytes from its specific host, the three‐spined stickleback (*Gasterosteus aculeatus*). Fish Shellfish Immunol. 35:1779–1787. 24036333 10.1016/j.fsi.2013.08.029

[evl3274-bib-0055] Schultz, E. T. , M. Topper , and D. C. Heins 2006. Decreased reproductive investment of female threespine stickleback *Gasterosteus aculeatus* infected with the cestode *Schistocephalus solidus*: parasite adaptation, host adaptation, or side effect? Oikos 114:303–310.

[evl3274-bib-0056] Simmonds, N. E. , and I. Barber 2016. The effect of salinity on egg development and viability of *Schistocephalus solidus* (Cestoda: Diphyllobothriidea). J. Parasitol. 102:42–46. 26418088 10.1645/14-701

[evl3274-bib-0057] Sprehn, C. G. , M. J. Blum , T. P. Quinn , and D. C. Heins 2015. Landscape genetics of *Schistocephalus solidus* parasites in threespine stickleback (*Gasterosteus aculeatus*) from Alaska. PLoS ONE 10:1–17. 10.1371/journal.pone.0122307 PMC4395347 25874710

[evl3274-bib-0058] Stefka, J. , V. Hypsa , T. Scholz , J. Štefka , V. Hypša , and T. Scholz 2009. Interplay of host specificity and biogeography in the population structure of a cosmopolitan endoparasite: microsatellite study of *Ligula intestinalis* (Cestoda). Mol. Ecol. 18:1187–1206. 19222754 10.1111/j.1365-294X.2008.04074.x

[evl3274-bib-0059] Steinel, N. C. , and D. Bolnick 2018. The fish adaptive immune response and its suppression by helminths. J. Immunol. 200:55–59.

[evl3274-bib-0060] Stuart, Y. E. , T. Veen , J. N. Weber , D. Hanson , M. Ravinet , B. K. Lohman , et al. 2017. Contrasting effects of environment and genetics generate a continuum of parallel evolution. Nat. Ecol. Evol. 1:1–7. 28812631 10.1038/s41559-017-0158

[evl3274-bib-0061] Stutz, W. E. , and D. I. Bolnick 2017. Natural selection on MHC IIβ in parapatric lake and stream stickleback: balancing, divergent, both or neither? Mol. Ecol. 26:4772–4786. 28437583 10.1111/mec.14158

[evl3274-bib-0062] Stutz, W. E. , O. L. Lau , and D. I. Bolnick 2014. Contrasting patterns of phenotype‐dependent parasitism within and among populations of threespine stickleback. Am. Nat. 183:810–825. 24823824 10.1086/676005

[evl3274-bib-0063] Tierney, J. F. , and D. W. T. Crompton 1992. Infectivity of plerocercoids of *Schistocephalus solidus* (Cestoda: Lingulidae) and fecundity of the adults in an experimental definitive host, *Gallus gallus* . J. Parasitol. 78:1049–1054. 1491297

[evl3274-bib-0064] Tierney, J. F. , F. A. Huntingford , and D. W. T. Crompton 1996. Body condition and reproductive status in sticklebacks exposed to a single wave of *Schistocephalus solidus* infection. J. Fish Biol. 49:483–493.

[evl3274-bib-0065] Vale, P. F. , A. J. Wilson , A. Best , M. Boots , and T. J. Little 2011. Epidemiological, evolutionary, and coevolutionary implications of context‐dependent parasitism. Am. Nat. 177:510–521. 21460572 10.1086/659002 PMC3725425

[evl3274-bib-0066] Viney, M. E. , E. M. Riley , and K. L. Buchanan 2005. Optimal immune responses: immunocompetence revisited. Trends Ecol. Evol. 20:665–669. 16701455 10.1016/j.tree.2005.10.003

[evl3274-bib-0067] Vrtílek, M. , and D. I. Bolnick 2021. Macroevolutionary foundations of a recently evolved innate immune defense. Evolution 75:2600–2612. 34347301 10.1111/evo.14316 PMC8488947

[evl3274-bib-0068] Weber, J. N. , M. Kalbe , K. C. Shim , N. I. Erin , N. C. Steinel , L. Ma , et al. 2017a. Resist globally, infect locally: a transcontinental test of adaptation by stickleback and their tapeworm parasite. Am. Nat. 189:43–57. 28035893 10.1086/689597

[evl3274-bib-0069] Weber, J. N. , N. C. Steinel , K. C. Shim , and D. I. Bolnick 2017b. Recent evolution of extreme cestode growth suppression by a vertebrate host. Proc. Natl. Acad. Sci. USA 114:6575–6580. 28588142 10.1073/pnas.1620095114 PMC5488926

[evl3274-bib-0070] Weber, J. N. , N. C. Steinel , F. Peng , K. C. Shim , B. K. Lohman , L. Fuess , et al. 2021. Evolution of a costly immunity to cestode parasites is a pyrrhic victory. bioRxiv 10.1101/2021.08.04.455160.

[evl3274-bib-0071] Wegner, K. M. , M. Kalbe , and T. B. H. Reusch 2007. Innate versus adaptive immunity in sticklebacks: evidence for trade‐offs from a selection experiment. Evol. Ecol. 21:473–483.

