# Supplementary material for: Population‐level variation in parasite resistance due to differences in immune initiation and rate of response
Source: Evol Lett. 2022 Feb 24;6(2):162–77. doi: 10.1002/evl3.274 (PMC8966477; doi:10.1002/evl3.274)
Supplement: Supplementary file 1 — Figure S1: Map of sampling locations for each of the study populations on Vancouver Island, Canada. Map curtesy of Vivid Maps 2021. Table S1: Least squared means and confidence intervals (from permuted t‐tests) for fibrosis response results from the laboratory injection experiment. [file EVL3-6-162-s002.pdf]

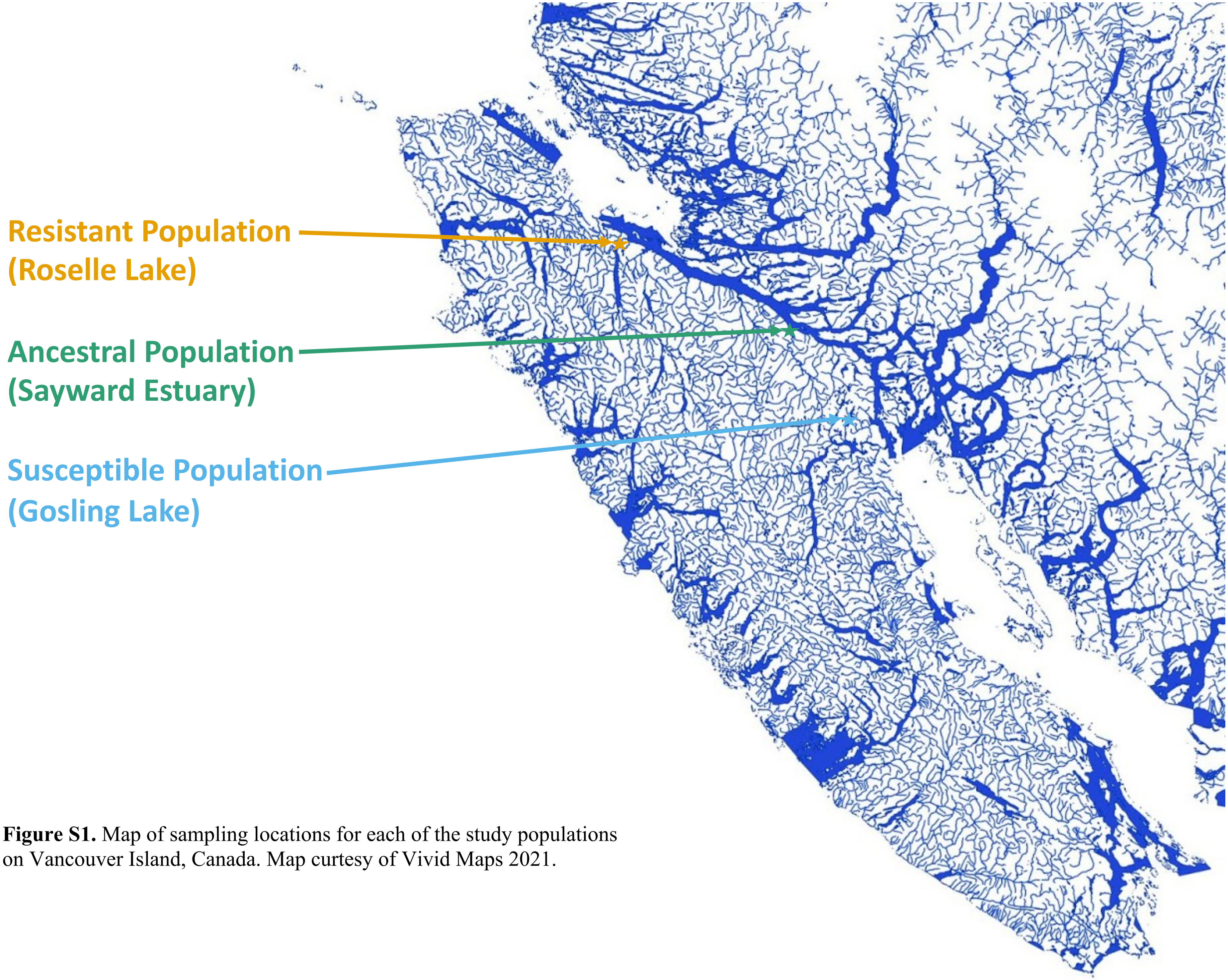

Figure S1. Map of sampling locations for each of the study populations on Vancouver Island, Canada. Map curtesy of Vivid Maps 2021.

Scoring Fibrosis: We scored fibrosis in the peritoneal cavity visually using a dissecting microscope as: 0 (no fibrosis), 1 (some fibrosis, organs do not move freely), 2 (fibrosis adhering organs together), 3 (organs adhered together and to the peritoneal wall), 4 (severe fibrosis, difficult to open peritoneal cavity). See supplemental video "Scoring Fibrosis in Fresh Stickleback Fish" for a visual description of these four scoring levels. Scoring fibrosis is inherently destructive, as the connective tissue is broken as the fish is opened and organs are moved to see if they are adhered. This scoring also had to be done quickly, as we wanted to remove organs for further gene expression analysis rapidly after the fish was euthanized. Given this, each fish was only scored by one person. This limitation led us to break a continuous phenotype into only 4 levels which were clearly definable and distinguishable. While this meant simplifying much variation, we specifically defined these levels such that we were confident we could distinguish between them.

Table S1. Least squared means and confidence intervals (from permuted t-tests) for fibrosis response results from the laboratory injection experiment. Broken up by time, population, and treatment. Exact values of least squared means and confidence intervals varied slightly depending on how models were constructed (question 1, 2, and 3 from the main text), but results were consistent.

	1 Day		10 Days		42 Days		90 Days	
	LSM	CI	LSM	CI	LSM	CI	LSM	CI
**Ancestral Population** *(Sayward)*								
PBS	0	*na*	0.00	-0.35, 0.35	0.08	-0.16, 0.32	0.00	-0.41, 0.41
Alum	0	*na*	2.82	2.47, 3.18	3.77	3.56, 3.99	4.00	3.67, 4.33
Tapeworm	0	*na*	0.11	-0.23, 0.44	0.04	-0.17, 0.26	0.00	-0.29, 0.29
Tapeworm+Alum	0	*na*	2.64	2.27, 3.00	3.68	3.43, 3.92	3.67	3.33, 4.00
**Susceptible Population** *(Gosling)*								
PBS	-0.01	-0.11, 0.08	-0.14	-0.53, 0.26	0.08	-0.47, 0.62	-0.16	-1.40, 1.08
Alum	0.00	-0.10, 0.10	2.27	1.88, 2.66	2.67	2.10, 3.24	3.12	2.36, 3.89
Tapeworm	-0.01	-0.10, 0.09	-0.06	-0.44, 0.33	0.14	-0.44, 0.72	0.49	-1.11, 2.11
Tapeworm+Alum	0.11	-0.01, 0.20	2.62	2.24, 3.01	3.20	2.63, 3.77	3.00	1.89, 4.10
**Resistant Population** *(Roselle)*								
PBS	0.04	-0.63, 0.72	0.16	-0.32, 0.65	0.13	-0.35, 0.62	0.00	-0.79, 0.79
Alum	1.24	0.56, 1.91	3.20	2.71, 3.69	2.71	2.22, 3.20	1.67	0.88, 2.46
Tapeworm	0.69	-0.00, 1.38	1.41	0.90, 1.92	0.57	0.10, 1.04	0.25	-0.23, 0.73
Tapeworm+Alum	1.69	1.01, 2.38	3.15	2.64, 3.66	2.30	1.80, 2.79	1.67	1.10, 2.22
